# Quality Enhancement Mechanism of Alkali-Free Chinese Northern Steamed Bread by Sourdough Acidification

**DOI:** 10.3390/molecules25030726

**Published:** 2020-02-07

**Authors:** Bowen Yan, Huayu Yang, Yejun Wu, Huizhang Lian, Hao Zhang, Wei Chen, Daming Fan, Jianxin Zhao

**Affiliations:** 1 State Key Laboratory of Food Science and Technology, Jiangnan University, Wuxi 214122, China; yanbowen2011@foxmail.com (B.Y.); 6170112163@stu.jiangnan.edu.cn (H.Y.); wuyejun@stu.jiangnan.edu.cn (Y.W.); zhanghao@jiangnan.edu.cn (H.Z.); chenwei66@jiangnan.edu.cn (W.C.); zhaojianxin@jiangnan.edu.cn (J.Z.); 2 School of Food Science and Technology, Jiangnan University, Wuxi 214122, China; 3 Wuxi Huashun Minsheng Food Co. Ltd., Wuxi 214218, China; lianhz666@163.com; 4 National Engineering Research Center for Functional Food, Jiangnan University, Wuxi 214122, China

**Keywords:** Chinese steamed bread, sourdough, acidification, gluten

## Abstract

Alkali was used to adjust the pH and neutralize the excess acids of dough in the processing of Chinese northern steamed bread (CNSB). However, extra alkali addition generally resulted in alkalic flavor and poor appearance. The aim of this work was to investigate the role of proofed dough pH on the texture of CNSB. Correlation analysis demonstrated that the pH value of proofed dough has a significant effect on the textural properties of CNSB. The mechanism studies found that gradual acidification of dough by lactic acid bacteria is a critical factor affecting the process. Conversely, chemical acidification weakened the texture property of products and reduced the dough rheology. Scanning electron microscope (SEM) analysis showed that fermentation with starter for 12 h produced a continuous and extensional protein network in the proofed dough. Furthermore, the decreasing pH of proofed dough increased the extractability of protein in a sodium dodecyl sulfate (SDS)-containing medium and the content of free sulfhydryl (SH). The structure and content of gluten, especially influenced by gradual acidification level, change the quality of the final product. It is a novel approach to obtain an alkali-free CNSB with excellent quality by moderate gluten adjustment.

Academic Editors: Adam Figiel and Anna Michalska

## 1. Introduction

Sourdough steamed bread is a traditional Chinese fermented food, which is usually produced either at home or in factories [1]. The texture of products differ significantly depending on consumer demand in different areas of China [2]. Therefore, steamed bread is divided into northern and southern steamed bread. Chinese northern steamed bread (CNSB) is firmer and more chewy than that in southern areas, and it is usually proofed with sourdough to improve the product quality [3]. However, in China, consumers are not used to eating steamed bread with a sour taste, which is significantly different from Western sourdough bread. During the preparation process, the appropriate dietary alkali (main ingredient Na_2_CO_3_) is usually added to neutralize the excess acidic components in the dough [4], but the amount of addition is mainly based on the subjective experience of bakers, which is difficult to control. Less addition led to unacceptably sour taste, but excessive addition resulted in products with a yellow appearance due to the combination of alkali and the isoflavone pigment in flour. Furthermore, the addition of alkali also seriously damages the nutrients in the dough, such as B vitamins, which greatly affect the product quality [5].

Acidification, the acids produced by microbial metabolism, is an important characteristic of sourdough fermentation [6]. The acidic environment plays an critical role in the formation of polymer structures, such as proteins, starch, and arabinoxylan, that are the major determinants of texture properties [7]. The optimum pH for sourdough is between 3.5 and 4.0 [8], which is suitable for activating the enzymatic activity of cereal protease. Previous studies proved that the pH-dependent cereal proteases and lactic acid bacteria-liberated proteases contribute to the depolymerization of gluten protein, which is an important aspect in the dough rheology and product quality [9].

Protein is degraded by both enzyme and acid during fermentation. This is consistent with the findings of Clarke [10] and Thiele [11], in which the activity of cereal protease is the main determinant of dough rheology under appropriately acidic conditions, and that differences in the acidification rates of strains result in significant differences in product quality.

The physical properties of wheat-based products are significantly related to the formation of gluten structure [12,13]. The effect of acids on the protein is mainly reflected in its swelling and solubility, which are related to the protein’s more positive net charge in an acidic environment [14]. An increase in intramolecular electrostatic repulsion causes the glutelin to unfold, exposing more hydrophobic groups. Strong electrostatic repulsion between molecules prevents the formation of new bonds, resulting in softer dough and a shorter mixing time [15]. The softness of gluten also promotes swelling and increases water absorption. Meanwhile, changes in acidification and fermentation time positively affect cereal enzyme activity. The best pH for flour proteases is in the acidic range, and protein hydrolysis increases in dough at a pH of 4 relative to non-acidified dough [16]. The prolongation of fermentation time can also increase the activity of cereal protease. The rheological consequence of gluten degradation appears to be a reduction in the elasticity and firmness of sourdough and steamed bread. Whether product size increases or decreases depends on the acidity of the dough and the morphology of the gluten network. The gluten network produced by sourdough fermentation increases gas retention due to physicochemical reactions, and softer and more ductile dough allows for greater expansion [17]. However, acidification and enzymatic hydrolysis may result in the complete degradation of high molecular weight gluten, leading to strong gluten softening. Although weaker gluten allows for greater expansion, it also reduces the dough’s gas retention. Therefore, the acidity levels of sourdough and proofed dough should be carefully controlled to ensure that the final product has the desired texture.

In recent years, researchers have focused on exogenous additives to improve the characteristics of CNSB [18,19,20]. Worthy of attention is the previous research by Wu [2] on adding different types of starter in CNSB. In this study, sourdough was fermented using *Lactobacillus plantarum* (*L.plantarum*), which is often found in samples of sourdough used to make CNSB, and the lactic acid bacteria proves to have excellent properties in CNSB, according to previous studies [2]. The purpose of this study is to investigate the optimum acidification of sourdough starter on the quality of CNSB to establish the relationship between the gluten of proofed dough and the texture property of steamed bread. The knowledge of gluten adjusting responsible for the quality of products will facilitate the industrial development of alkali-free steamed bread.

## 2. Results and Discussion

### 2.1. Effects of Acidification Level on Textural Properties and Sensory Evaluation of Northern Steamed Bread

**Figure 1:**
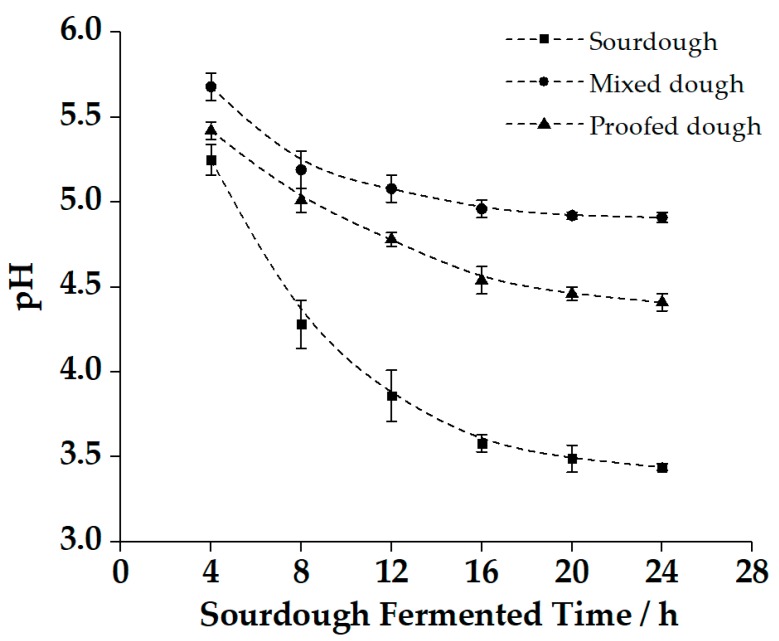
Final pH of sourdough, mixed dough, and proofed dough. The mixed dough and proofed dough were prepared by corresponding sourdough with different fermentation times: 4–24 h. All data are the means ± standard deviation.

To explore the effect of sourdough acidification on the quality of CNSB, pH changes of dough during CNSB processing were investigated. Dough proofed with sourdough starter was performed every 4 h, and the pH values of sourdough starter, mixed dough, and proofed dough were analyzed, as shown in Figure 1. A significant reduction in pH was observed from 4 h to 16 h of fermentation in the mixed dough and proofed dough. It can be determined that the addition of sourdough starter with different fermentation times contributed to the pH changes during CNSB processing, and the trend of change was consistent, which had a direct impact on the pH of the final product.

**Figure 2:**
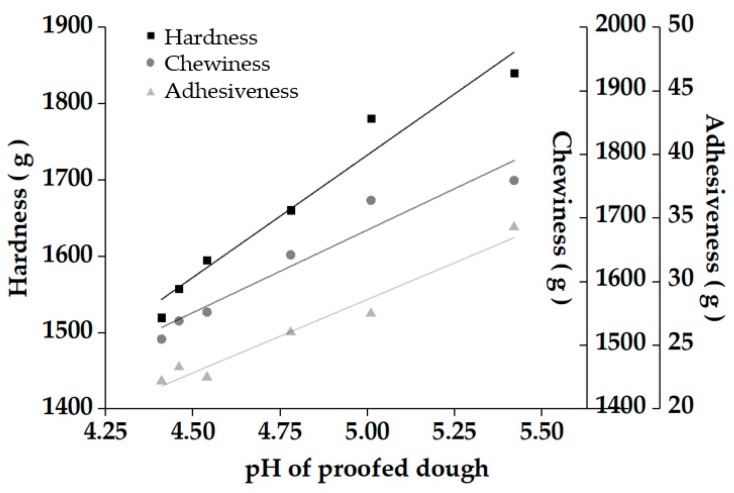
Correlation analysis between the final pH of proofed dough and the texture of steamed bread: R^2^ = 0.95821 (hardness); R^2^ = 0.93295 (chewiness); R^2^ = 0.96348 (adhesiveness).

**Figure 3:**
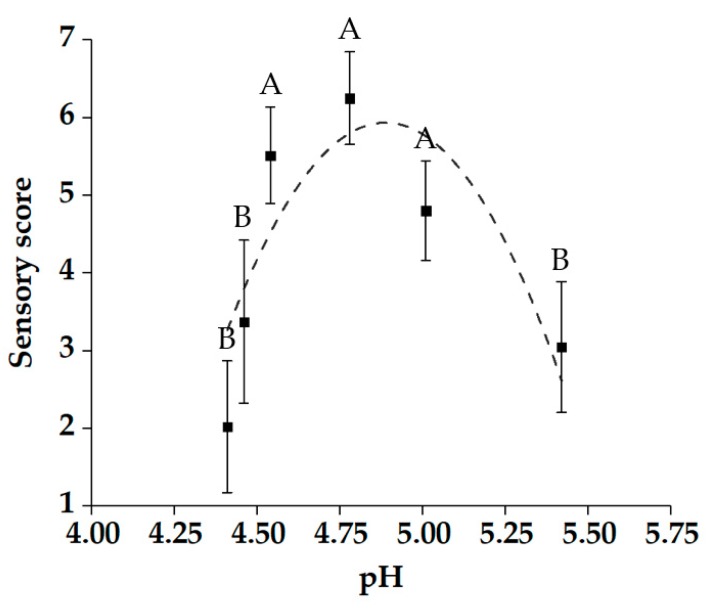
Effects of the final pH of proofed dough on steamed bread sensory evaluation. All data are the means ± standard deviation. Sensory score: 1: very unacceptable; 2: unacceptable; 3: mildly unacceptable; 4: neither unacceptable nor acceptable; 5: mildly acceptable; 6: acceptable; and 7: very acceptable.

Texture is important for evaluating the quality of steamed bread, as the previously reported hardness, chewiness, and adhesiveness were generally detected as the key indicators for texture evaluation [21]. From the results in Table 1, the addition of sourdough reduced the hardness, chewiness, and adhesiveness of the steamed bread, which resulted in a softer texture. In addition, the correlation analysis between the pH value of proofed dough and the texture of steamed bread revealed that pH value is positively correlated with the major texture indicators of steamed bread (R^2^ = 0.9330–0.9635) (Figure 2). However, the level of texture indicators cannot directly explain the consumer acceptability of steamed breads, and no previous study has determined an optimal value for steamed breads so far. Therefore, more effective evaluation methods must be used to further explore. Sensory evaluation was performed by a consumer panel to further verify the effect of final pH levels on the quality of steamed bread. The consumer panel was asked to score the samples according to the degree of preference. The results showed that the relationship between pH value and sensory score was not linear, which indicated that the adjustment of sourdough acidity is related to the taste of steamed bread. Sensory scores increased and then dropped significantly in value when the pH level decreased (Figure 3). Previous studies reported that the pH value of sourdough fermentation has an optimal range under the influence of many factors [8], and the results of sensory evaluation confirmed a similar effect in the production of steamed breads. The results were in line with our previous hypothesis that a high-scoring region may correspond to moderate texture characteristics, which existed at a moderate acidification level for the quality of CNSB. In addition, based on the results of sensory evaluation, the data obtained by the texture experiment correspond to the sensory score for concluding the range of texture parameters with high consumer acceptance, which is not only conducive to the quality evaluation, but also facilitates the industrial development of alkali-free steamed bread.

**Figure 4:**
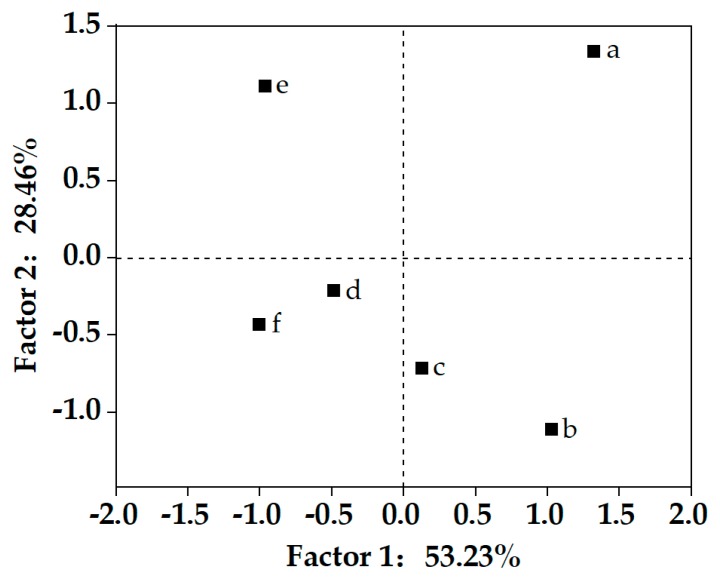
Principal component analysis (PCA) of Chinese steamed bread with different sourdough fermentation times: (a) 4 h; (b) 8 h; (c) 12 h; (d) 16 h; (e) 20 h; (f) 24 h.

To further explore whether there was a tendency for clustering of steamed breads prepared by culture starters fermented with different times, the textural and sensory parameters were subjected to principal component analysis (Figure 4). The steamed bread was divided into three clusters according to the final pH value of the proofed dough: approximately 5.42 ± 0.05, 4.78 ± 0.04 to 5.01 ± 0.07, and 4.54 ± 0.08 to 4.41 ± 0.05. The proofed dough with a pH of 4.78 ± 0.04 scored higher for sensory quality than the other products. The results suggest that the moderate acidification level of proofed dough has positive effects on the textural properties and sensory quality of CNSB. Based on the findings of the principal component analysis, the proofed dough with starters for 4 h, 12 h, and 20 h, which showed significant differences and represented different clusters of steamed bread, were selected for further experiments.

### 2.2. Effects of Chemical and Biological Acidification on Textural Properties and Rheological Characteristics

**Figure 5:**
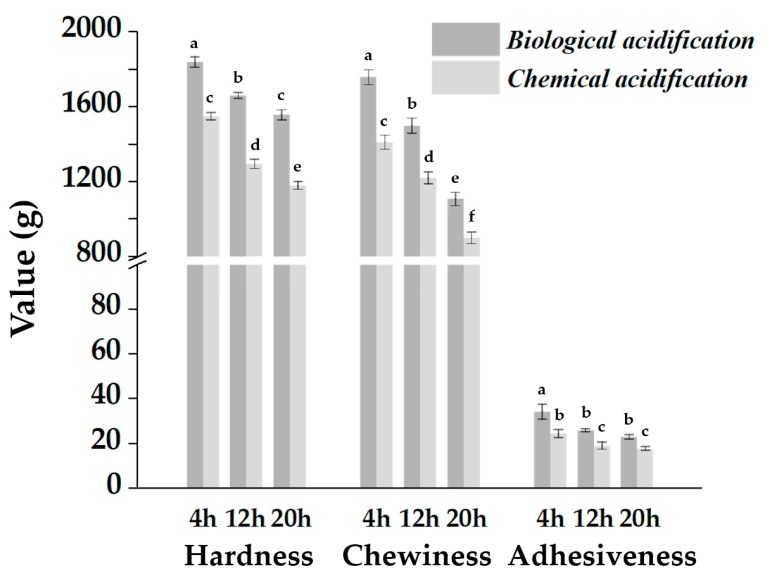
Texture of Chinese steamed bread with different acidification methods. Data are presented as means ± standard error of the mean.

Chemical and biological acidification methods were compared to determine the effect of changes in pH on the texture of the steamed bread. Steamed bread produced by chemical acidification resulted in weakened hardness, chewiness, and adhesiveness as compared to those proofed with sourdough (Figure 5); this indicates that the final pH value of proofed dough is not the key factor for textural improvement. Chemical acidification damaged the gluten network structure of the dough due to the rapid reduction in pH level during proofing. As previously reported by Kopec’ [22], sourdough fermentation involves gradual acidification through microbial metabolism. The decreasing pH level affects the physicochemical properties of gluten by activating the protease in cereal flour and changing the rheological properties and microstructure of the dough. However, the exogenous addition of organic acids leads to instant acidification, which has a negative effect on the structure of dough.

**Figure 6:**
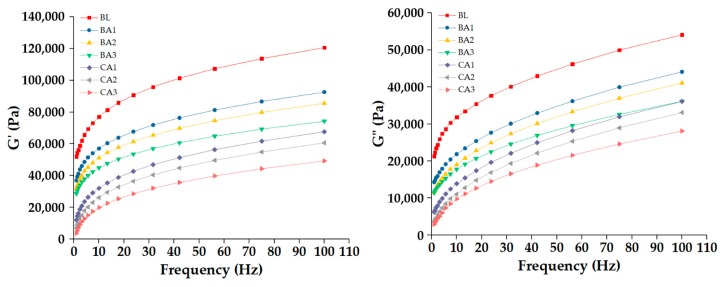
Effect of acidification methods on dough elastic modulus (G’) and viscous modulus (G”): (BL) blank group; (BA1) bio-acidification for 4 h; (BA2) bio-acidification for 12 h; (BA3) bio-acidification for 20 h; (CA1) chemical acidification to BA1 pH level; (CA2) chemical acidification to BA2 pH level; (CA3) chemical acidification to BA3 pH level.

To further investigate the effect of acidification on the elasticity and viscosity of proofed dough, the rheological properties were analyzed (Figure 6). The decreasing pH reduced the elastic and viscous modulus, which indicated that the low pH degraded the gluten protein and weakened the structure of dough. Compared to the proofed dough with sourdough starter, the chemical acidified samples exhibited less elasticity and viscosity because the rapid degradation of gluten protein damaged the network structure of the dough. The results related to the weakening of intermolecular or intramolecular disulfide bonds [19]. Clarke [10] proved that acidification resulted in a more positive net charge in the dough system, it may be the main reason for the stickiness and softness of dough. However, the increased electrostatic repulsion contributed to protein solubility, which led to the expansion of gluten molecules and the exposure of hydrophobic groups. These changes limited the formation of new chemical bonds and weakened the structure of the gluten. Ketabi et al. [23] also found that Lactobacillus can metabolize fructan, which as a polymer positively affects dough rheological properties during fermentation.

### 2.3. Microstructure of Proofed Dough

**Figure 7:**
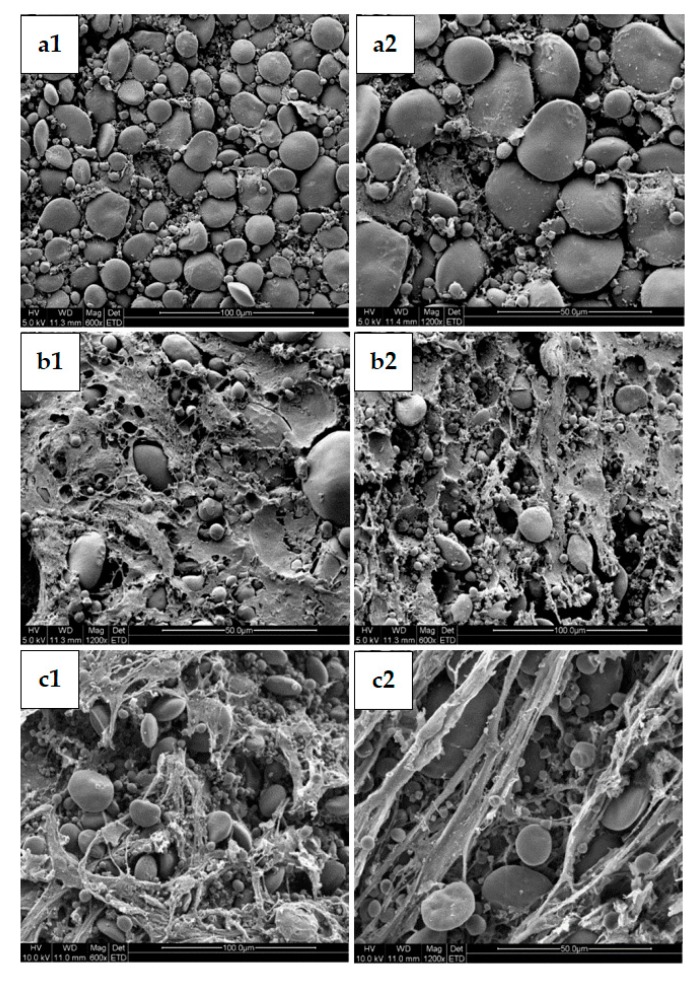
Scanning electron micrographs (SEMs) on proofed dough with different acidification levels: (**a1**,**a2**) added 4 h fermented sourdough; (**b1**,**b2**) added 12 h fermented sourdough; (**c1**,**c2**) added 20 h fermented sourdough.

The above results reveal that changes in pH levels led to significant differences in product texture and dough rheological properties. The microstructure of proofed dough was also observed by SEM. The starch granules were round or oval and the gluten proteins were flaky or silky. The results indicate that proofed dough with starter for 4 h showed conspicuous starch granules and a firm and dense gluten network (Figure 7(a1,a2)). However, dough proofed with starter for 12 h had an improved the gluten network structure, enabling its continuous structure to be clearly observed. The starch granules were embedded in the gluten and combined rigidly (Figure 7(b1,b2)). Consistent with previous results, the best sensory evaluation of product was obtained by adding 12 h starter owing to the acidification adjustment which results in the depolymerization of macromolecule proteins and the formation of gluten proteins with a fibrous structure and greater continuity [23]. However, after fermentation with sourdough for 20 h, the gluten structure was broken and discontinuous (Figure 7(c1,c2)). The extremely low pH level and long fermentation time led to the excessive degradation of large complex polymers; previous studies show that it leads to the destruction of intermolecular disulfide bonds between glutenin and gliadin and that starch grains are exposed [24]. The pH level was too low to improve the ability of the dough to hold gas, resulting in products with low hardness and poor elasticity. Therefore, it was extremely important for adjusting sourdough acidification levels to obtain the desired gluten protein of proofed dough.

### 2.4. Extractability of Gluten Protein

Wheat gluten quality was an important determinant of dough rheological properties and the suitability of products for processing, and it was dependent on the degree of cross-linking of proteins [25]. The extractability of proteins in a sodium dodecyl sulfate (SDS)-containing medium was found to be a good indicator of the degree of cross-linking of wheat gluten [26]. The extraction rates of dough at different stages of processing were compared by adding sourdough starters for 4 h, 12 h, and 20 h. 

A slight change was observed in the extractable protein during mixing (Table 2). The results suggest that depolymerization decreased during the mixing process. Lower pH value of dough increased the solubility of the protein in the proofed dough (Table 2). The pH level was lower than gluten’s isoelectric point, and the net positive charge helped to produce electrostatic repulsion between the protein molecules, which increased the rate of extraction of the gluten protein [27]. The acidic environment induced the degradation of the macromolecule SDS-insoluble protein to small soluble molecules. The lower pH which resulted from microbial fermentation also activated wheat endogenous proteases and strain-specific proteolytic enzymes, further dissociating the proteins in the dough [28]. Attention should be paid to the changes in disulfide bond content during cross-linking, which determines the morphology of the protein [29]. The improvement in the rheological properties of the steamed bread and the reduction in its hardness were mainly due to the depolymerization of glutenin macromolecules, which made the cross-linking structure uniform and regular [30]. During proofing, hydrogen peroxide produced by *L. plantarum* promoted the oxidation of free sulfhydryl groups to form disulfide bonds and linked the hydrophobic amino acids in the molecule. The generation of an α-helice and β-sheet structure improved the elasticity of the dough [31]. The increasing of gliadin content contributed to the ductility of dough (Table 2) and gave it more resilience during stretching. Excessive stickiness made dough difficult to process. Similarly, the excessive degradation of glutenin macropolymer destroyed the gluten network and weakened the texture of the products.

The extractability of protein was significantly reduced (*p* < 0.05) after steaming, relative to the previous two stages (Table 2), which indicates that the protein aggregated. When fully hydrated gluten was heated above 75 °C, both gliadin and glutenin could be incorporated into the protein network structure [32]. The protein was susceptible to cross-linking through the formation of disulfide bonds under high temperature conditions, forming complex macromolecular proteins through the random interleaving of space and interior, and thereby substantially reducing extractable SDS-soluble protein. However, we found that the extractability of the gluten increased slightly with acidification time (Table 2). The acidification degraded the SDS-insoluble protein during sourdough fermentation, but the decreasing pH value was not conducive to the formation of disulphide (SS) cross-linking, which was most likely to occur under alkaline conditions (cysteine’s pKa ≈ 8.5) [33].

### 2.5. Free sulfhydryl (-SH) Changes Induced by Processing

**Figure 8:**
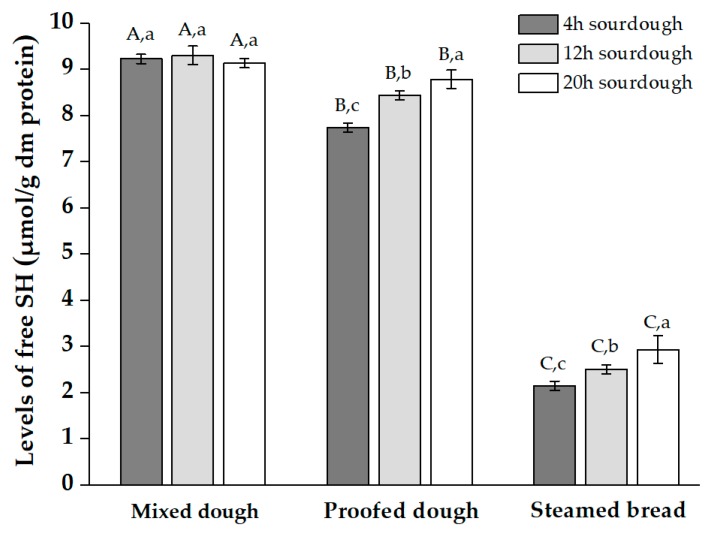
The level (μmol/g dm protein) of free sulfhydryl (SH) of mixed dough, proofed dough, and steamed bread with different acidification levels of sourdough. The three results in each group were added 4 h, 24 h, and 36 h fermented sourdough, respectively.

Free sulfhydryl content reflected the changes of disulfide bonds. A strong link was found between disulfide bond content and gluten protein structure [34]. The level of free SH decreased significantly after fermentation and steaming (Figure 8), which indicates that heating led to the formation of disulfide bonds and contributed to the structure of the steamed bread. pH level had little effect on the free SH content during the mixing stage, corresponding to slight changes in protein extractability. However, the free SH level increased as pH value decreased during fermentation and steaming (Figure 8). Acidification may have weakened the oxidation of the free SH and SH–SS exchange reactions [35]. The structure of the complex gluten network formed by SS cross-linking was weakened, which possibly manifested in the depolymerization of glutenin and simplification of complex network structures. These processes formed more SDS-soluble protein and provided more free SH for the system, which corresponded to increased protein extractability and a higher free SH level [26]. In addition, the decreasing pH value also reduced the occurrence of SS cross-linking. During the production of CNSB, sufficient protein aggregates were required to form a gluten scaffold, but excessive polymerization leads to unsatisfactory hardness and chewiness [36]. Briefly, gluten levels should maintain within an appropriate range during processing. Remarkably, the metabolites produced by *L. plantarum* during fermentation included hydrogen peroxide and glutathione. The oxidation of hydrogen peroxide and the free SH in the reduced glutathione may have interfered with our results, but their effects are ignored due to their low content and decomposability under heat treatment.

## 3. Materials and Methods

### 3.1. Materials

Wheat flour (Wudeli Flour Group Co., Ltd., Wuxi, China) and baker’s yeast (Angel Yeast Co., Ltd., Yichang, China) were purchased from the local supermarket in Wuxi. The moisture, protein (N × 5.7), and ash contents of wheat flour were 11.57 ± 0.02%, 13.19 ± 0.04%, and 0.37 ± 0.01%, respectively, and they were determined according to AACC Approved Methods 44-15A, 46-12, and 08-01, respectively [37]. The freeze-dried powder of *Lactobacillus plantarum CCFM8610* (*L.plantarum*) was obtained from Jiangnan University (Wuxi, China).

### 3.2. Preparation of Chinese Northern Steamed Bread (CNSB)

Batches, 100 g of wheat flour, 50 g of sterile distilled water, and 0.14 g of *L. plantarum* freeze-dried powder (3 × 10^10^ cfu/g) were weighed and mixed to prepare the sourdough, then proofed at 30 °C and 85% relative humidity for 4 h, 8 h, 12 h, 16 h, 20 h, and 24 h, respectively. CNSB was prepared as the method reported by Huang [38] with minor modification. The basic recipe of CNSB involves 400 g of wheat flour, 180 g of sterile distilled water, 1.2 g of baker’s yeast, and 80 g of sourdough, which were processed by using a mixer (KM080, Kenwood, London, UK). After mixing, the dough was divided into 100 g/piece for rounding and proofing at 35 °C and 80% relative humidity for 60 min. The proofed buns were steamed for 20 min. Samples of dough at every stage (mixed, proofed, and steamed) were immediately frozen in liquid nitrogen and freeze-dried. Samples of each group were processed in duplicate.

Chemically acidified steamed bread: after dividing the dough into 100 g/piece, different amounts of organic acids (lactic acid (88%):acetic acid (99.5%) = 4:1, *v*/*v*) were added and kneaded by hand 20 times to make the pH value of the steamed bread the same as the biologically acidified steamed bread.

### 3.3. Determination of pH Value

The pH value of dough at different process stages was measured as previously reported by Yan Bowen et al. [39]. Sample aliquots (10 g) were homogenized with 90 mL of sterile distilled water for 10 min with a magnetic stirrer (IKA basic 2 RH, Staufen, Germany). All of the tests were performed in triplicate.

### 3.4. Sensory Evaluation

The sensory evaluation of the samples was performed following the previously described method by Fu [40] with minor modification. The sensory studies were reviewed for their adherence to ethical guidelines and approved by the Research Ethics Board at Jiangnan University. All panelists were selected and trained by GB/T 16291.1-2012, “Sensory analysis–General guidance for the selection, training and monitoring of assessors–Part 1: Selected assessors,” which is a national standard approved by the Standardization Administration of the People’s Republic of China. The steamed bread prepared with sourdough was steamed for 20 min and then cooled at room temperature for 45 min before being subjected to sensory evaluation. All of the samples were evaluated within 60 min of preparation.

Overall, 80 sensory consumer panelists were recruited randomly at the Department of Food Science and Technology, Jiangnan University. The number of male and female panelists was about equal. Most of the panelists (76 panelists) were 18–30 years old. A majority of panelists came from northern China and consumed steamed bread more than 2–3 times per week, and they were all consumers of traditional Chinese steamed bread. The panelists received six encoded samples (added sourdough with fermentation time of 4 h, 8 h, 12 h, 16 h, 20 h, and 24 h, respectively) and a questionnaire as well as instructions for the evaluation of samples. Samples were randomly assigned to each panelist. The samples were presented to the assessors blind so that the panelists did not know which sample they were evaluating. Water was provided to cleanse the palate to prevent the influence between samples. The appearance, viscosity, elasticity, and taste of the samples were evaluated on a 7-point hedonic scale, which corresponds to 1–7 points: 1: very unacceptable; 2: unacceptable; 3: mildly unacceptable; 4: neither unacceptable nor acceptable; 5: mildly acceptable; 6: acceptable; and 7: very acceptable. The final sensory score is the average of all indicators.

### 3.5. Textural Profile Analysis (TPA) of CNSB

The steamed bread samples were subjected to TPA using a textural analyzer equipped with a P35 pressure plate probe. The specific test conditions were as follows: pre-test speed: 1 mm/s; test speed: 1.7 mm/s; post-test speed: 10 mm/s; and compression rate: 40%. Hardness, chewiness, and adhesiveness were measured using a TPA curve.

### 3.6. Rheological Properties of Dough

To maintain the stability of the dough samples, yeast was not added during preparation, and antibiotics were added to inhibit the growth of lactic acid bacteria in the dough after mixing. The chemical acidification group comprised 0.02% erythromycin, 0.02% cycloheximide, and 0.004% chloromycetin. The parameters were set as described by Huang [41]. Rheometer frequency was set to 1.0 Hz, with a frequency range of 0.1–100 Hz; 0.4 cm parallel plates were positioned at 1 mm intervals; and the temperature was 30 °C.

### 3.7. Size-Exclusion Chromatography (SEC) High-Performance Liquid Chromatography (HPLC) Analysis of Proteins

Protein extraction and SEC-HPLC were conducted as described by Thiele [11], with some modifications. The lyophilized samples were subjected to 1:10 extraction with buffers (50 mM sodium phosphate, 1.5% sodium dodecyl sulfate (SDS), pH 6.9) and shaken for 30 min. The protein in the first extraction residue was dissolved in a buffer (4% dithiothreitol (DTT), 50 mM sodium phosphate, 1.5% SDS, pH 6.9). The SDS extracts and the SDS-DTT extracts were applied to a Superdex 200 column coupled with a Superdex Peptide column and a Superdex 200 column (both from Amersham Bioscienes, Uppsala, Sweden), respectively, with fractionation molecular weight ranges of 100 to 5 × 10^6^ and 104 to 5 × 10^6^, respectively. The samples were eluted at room temperature with a buffer containing 0.1% SDS and 20% acetonitrile in 50 mM sodium phosphate buffer (pH 7.7) at a flow rate of 0.4 mL/min. Ultraviolet detectors were set to 210 nm and 280 nm. The 280 nm trace was used to identify low molecular weight components to prevent the interference of lactic acid or other non-protein carboxyl compounds. The results were divided into three fractions by the lowest point between peaks, corresponding to the level of extractable glutenin in the SDS buffer, the level of SDS-extractable gliadin, and the level of SDS-extractable albumin and globulin from front to back.

### 3.8. Determination of Free Sulfhydryl (SH) Group Content

The total content of free SH was determined using the method specified by Wang [34]. The following solvents were used: Tris-glycine-Ethylene Diamine Tetraacetic Acid (EDTA) buffer (10.4 g Tris, 6.9 g glycine, and 1.2 g EDTA per liter, pH 8.0, denoted as TGE), Ellman’s reagent (5,5′-dithiobis-2-nitrobenzoic acid) in TGE (4 mg/mL), and 2.5% SDS in TGE (SDS-TGE). Then, 40 mg samples were added to 4 mL of SDS-TGE reagent, respectively, and mixed thoroughly for 30 min with vortexing every 10 min. Next, 0.04 mL of Ellman’s reagent was added, followed by thorough mixing for 30 min. The absorbance of the supernatant was measured at 412 nm. The blank lacked Ellman’s reagent and the samples. The absorbance values were converted to amounts of free SH using a calibration curve with reduced glutathione.

### 3.9. Scanning Electron Microscopy (SEM)

The dough microstructure was observed by SEM. The dough samples were fixed with a 2.5% glutaraldehyde solution (*v/v*) for 4 h, and the dough was rinsed three times with 0.1 mol/L phosphate buffer and eluted once with 20%, 40%, 60%, 70%, and 80% ethanol solution (*v/v*), respectively. Next, the dough was eluted three times with 100% ethanol for an average of 20 min per elution. The acceleration voltage was set to 1.0 kV, an ion sputtering gold spray was applied for 4 min, and the samples were freeze-dried and placed under SEM for observation.

### 3.10. Statistical Analysis

Analysis of variance (ANOVA) was performed using the software package SPSS 19.0 (SPSS Inc., Chicago, IL, USA). One-way ANOVA, principal component analysis, and Duncan’s multiple-range test were conducted. A significance level *of p* < 0.05 was used to determine the significance of the differences between the samples. Principal component analysis (PCA) was performed on MetaboAnalyst to analyze dissimilarities among the samples in terms of their textural profile and sensory scores [6].

## 4. Conclusions

The results show that pH value is positively correlated with the hardness, chewiness, and adhesiveness of CNSB, with a moderate range for product sensory properties. This phenomenon not only relates to the slow rate of acidification by *L. plantarum* during fermentation, but also to the gluten level of proofed dough. Reducing the pH of the proofed dough increased both the extractability of protein in an SDS-containing medium and the content of free SH, resulting in changes to the rheological properties and microstructure of the proofed dough, which in turn affected the texture of the product. In conclusion, moderate gluten adjustment of proofed dough plays an important role in developing the alkali-free CNSB with desired textural and sensory properties. The mechanisms exploration from this study can be referenced by food technologists for standardizing the production methods for alkali-free CNSB in the future.

## Figures and Tables

**Table 1:** Effect of sourdough fermented time on the texture of Chinese northern steamed bread.

Sourdough Fermented Time	Final pH of Proofed Dough	Hardness (g)	Adhesiveness	Springiness	Chewiness	Stickiness
4 h	5.42 ± 0.05 a	1840.3 ± 27.8 a	34.3 ± 4.3 a	0.93 ± 0.01 a	1759.3 ± 16.0 a	2000.5 ± 29.0 a
8 h	5.01 ± 0.07 b	1780.8 ± 12.4 b	27.5 ± 0.9 b	0.92 ± 0.01 a	1728.1 ± 25.6 b	1891.8 ± 16.6 b
12 h	4.78 ± 0.04 c	1660.8 ± 15.8 c	26.0 ± 0.7 bc	0.93 ± 0.02 a	1642.5 ± 31.8 c	1799.3 ± 27.1 c
16 h	4.54 ± 0.08 d	1595.0 ± 1.5 d	22.5 ± 0.7 cd	0.93 ± 0.01 a	1552.4 ± 19.0 d	1680.6 ± 23.5 d
20 h	4.46 ± 0.04 e	1558.0 ± 26.9 e	23.3 ± 1.0 cd	0.94 ± 0.03 a	1538.6 ± 15.0 e	1642.8 ± 5.4 e
24 h	4.41 ± 0.05 f	1520.0 ± 11.0 f	22.2 ± 0.6 d	0.93 ± 0.02 a	1510.0 ± 21.1 f	1620.8 ± 30.1 f

a–f: represents the significant difference within the columns.

**Table 2:** Protein extractability in a sodium dodecyl sulfate (SDS)-containing buffer.

Stage	Sourdough Fermented Time (h)	pH	SDS Rxtractable Glutenin (%)	SDS Extractable Gliadin (%)	SDS Extractable Protein (%)
Mixed	4	5.68 ± 0.08 a	23.03 ± 0.36 b	46.76 ± 0.25 e	69.78 ± 0.61 d
	12	5.08 ± 0.08 c	20.38 ± 0.44 c	47.84 ± 1.03 de	68.22 ± 1.47 e
	20	4.92 ± 0.02 d	18.65 ± 0.95 d	51.24 ± 0.78 b	69.89 ± 1.83 d
Proofed	4	5.42 ± 0.05 b	23.45 ± 0.47 b	48.30 ± 0.56 d	71.75 ± 0.71 c
	12	4.78 ± 0.04 e	25.52 ± 0.76 a	49.61 ± 0.47 c	75.13 ± 1.23 b
	20	4.46 ± 0.04 f	22.61 ± 0.4 b	53.68 ± 0.62 a	76.29 ± 1.02 a
Steamed	4	-	0.87 ± 0.07 e	9.84 ± 0.15 g	10.71 ± 0.22 h
	12	-	1.19 ± 0.02 e	10.65 ± 0.06 fg	11.84 ± 0.08 g
	20	-	1.90 ± 0.11 e	11.63 ± 0.23 f	13.52 ± 0.34 f

Data are expressed as mean ± standard deviation (*n* = 2). Means in the same column with different small superscript letters indicate significant difference at *p* < 0.05. Protein extractability (%) in SDS is always calculated from the corresponding peak area and expressed as a percentage of peak area of wheat flour under reducing conditions, which represents the total SDS-extractable protein in the flour since reduced gluten proteins are completely extractable. a–f: represents the significant difference within the columns.
